# Do older English adults exhibit day-to-day compensation in sedentary time and in prolonged sedentary bouts? An EPIC-Norfolk cohort analysis

**DOI:** 10.1371/journal.pone.0224225

**Published:** 2019-10-25

**Authors:** Dharani Yerrakalva, Katrien Wijndaele, Samantha Hajna, Kate Westgate, Kay-Tee Khaw, Nick Wareham, Simon J. Griffin, Soren Brage

**Affiliations:** 1 Department of Public Health and Primary Care, University of Cambridge School of Clinical Medicine, Cambridge, United Kingdom; 2 MRC Epidemiology Unit, University of Cambridge, School of Clinical Medicine, Cambridge, United Kingdom; Linneaus University, SWEDEN

## Abstract

**Introduction:**

Compensatory behaviours may be one of the reasons for the limited success of sedentary time interventions in older adults, but this possibility remains unexplored. Activity compensation is the idea that if we change activity levels at one time we compensate for them at a later time to maintain a set point. We aimed to assess, among adults aged ≥60 years, whether sedentary time and time spent in prolonged sedentary bouts (≥30 mins) on one day were associated with sedentary time and time spent in prolonged sedentary bouts (≥30 mins) on the following day. We also sought to determine whether these associations varied by sociodemographic and comorbid factors.

**Methods:**

Sedentary time was assessed for seven days using hip-worn accelerometers (ActiGraph GT1M) for 3459 adults who participated in the EPIC-Norfolk Study between 2004 and 2011. We assessed day-to-day associations in total and prolonged bouts of sedentary time using multi-level regressions. We included interaction terms to determine whether associations varied by age, sex, smoking, body mass index, social class, retirement, education and comorbid factors (stroke, diabetes, myocardial infarction and cancer).

**Results:**

Participants (mean age = 70.3, SD = 6.8 years) accumulated 540 sedentary mins/day (SD = 80.1). On any given day, every 60 minutes spent in sedentary time was associated with 9.9 extra sedentary minutes on the following day (95% CI 9.0, 10.2). This association was greater in non-retired compared to retired participants (non-retired 2.57 extra minutes, p = 0.024) and in current compared to former and never-smokers (5.26 extra mins for current vs former; 5.52 extra mins for current vs never, p = 0.023 and 0.017, respectively). On any given day, every 60 minutes spent in prolonged bouts was associated with 7.8 extra minutes in these bouts the following day (95% CI 7.6, 8.4). This association was greater in older individuals (0.18 extra minutes/year of age, 95% CI 0.061, 0.29), and for retired versus non-retired (retired 2.74 extra minutes, 95% CI 0.21, 5.74).

**Conclusion:**

Older adults did not display day-to-day compensation. Instead, individuals demonstrate a large stable component of day-to-day time spent sedentary and in prolonged bouts with a small but important capacity for positive variation. Therefore older adults appear to be largely habitual in their sedentary behaviour. Strategies to augment these patterns may be possible, given they may differ by age, smoking, and working status.

## Introduction

Sedentary time is a risk factor for a multitude of adverse health outcomes including type 2 diabetes, cardiovascular disease, cardiovascular mortality, cancer, cancer mortality and all-cause mortality[1–4]. Since older adults spend significant amounts of time being sedentary[5], designing strategies to reduce their sedentary time is important. Prolonged sedentary bouts appear to be particularly harmful to metabolic health [6–9]. Therefore prolonged sedentary time may represent a more important target for interventions than focusing only on reducing total sedentary time. Very few intervention studies have targeted prolonged bouts in this age group.

There is little evidence that interventions achieve sustained decreases in total sedentary time beyond 12 months[10]. Compensatory behaviours may be one of the reasons for this limited success[11]. Activity compensation is the idea that if we change activity levels at one time we compensate for them at a later time to maintain a set point. This is commonly referred to as the ‘Activity-stat’ hypothesis. Though it is postulated that compensation happens across the activity spectrum, the literature has focussed on compensation in moderate-to-vigorous physical activity (MVPA) and neglected sedentary behaviour[11].

Day-to-day sedentary time compensation would be indicated by negative associations between sedentary time on one day and the next day (decreased sedentary time on one day being associated with increases in sedentary time the next day). Similarly, it is important to elucidate whether day-to-day compensation occurs for time spent in prolonged sedentary bouts. This would be seen if decreased time in prolonged sedentary bouts on one day was associated with increased sedentary time in prolonged bouts on the next day. Conversely others have postulated that day-to-day ‘activity synergy’[12] may exist (i.e. positive associations between activity levels from one day to the next).

It has been suggested that compensation is biologically plausible and might occur because of homeostatic mechanisms through neural and hormonal feedback [11,13,14], muscle soreness, compensatory health beliefs, fear of overexertion, deficient motivation, or perceived time constraints [15]. Sedentary compensation could be triggered by naturally occurring day-to-day fluctuations (e.g. food shopping in the supermarket on one day, so sitting more the next day in compensation) which we might see in observational studies. It could also be observed in a structured, planned experimental intervention (e.g. seeing a health professional for advice and doing advised standing/walking one day, which is compensated with sitting more the next day). Observing whether day-to-day fluctuations occur is important as it may suggest we need to take compensation into account regardless of the nature or intensity of a future intervention and further, that compensation itself could be a target for future interventions. Characterising the nature and extent of compensation would also be important for designing interventions. For example, it is possible that in order to trigger a compensatory response a tolerance threshold might need to be reached. If this is the case, current interventions could be modified to within a tolerance range in order to avoid triggering compensation[11].

To our knowledge sedentary time compensation has not been investigated in older adults. Given that older adults spend large amounts of their day sedentary [16] and efforts to reduce time in this behaviour over the longer term have been unsuccessful,[10] it is important to elucidate whether compensation exists and if it is one of the barriers we need to address in future interventions. In a cohort of English older adults, we aimed to assess whether older adults’ sedentary time on one day was associated with sedentary time on the following day. This analysis was undertaken to determine whether day-to-day sedentary time compensation exists. Our secondary aims were to assess whether time in prolonged sedentary bouts (≥30 mins duration) on one day was associated with these behaviours on the following day and whether the day-to-day sedentary time or the day-to-day time in prolonged sedentary bout associations varied by sociodemographic and comorbid factors.

## Methods

### Study sample

The European Prospective Investigation of Cancer (EPIC) Norfolk Study has been described in detail elsewhere [17]. In brief, EPIC Norfolk is a large prospective cohort of over 25,500 adults living in Norfolk (England, UK), who undertook baseline assessments between 1993–1997. Participants were similar to the national population sample studied in the Health Survey of England in terms of anthropometry, serum lipids and blood pressure[18]. Participants in the 3^rd^ Health Check, conducted between 2004 and 2011, were invited to wear an accelerometer (ActiGraph GT1M, Pensacola, USA)[19,20]. Ethical procedures in this study were approved by the Norfolk Local Research Ethics and East Norfolk and Waveney NHS Research Governance Committee (05/Q0101/191). Participants gave signed informed written consent. The EPIC-Norfolk Approval Board specifically approved access to data for use in this study. The present analysis was restricted to participants aged ≥ 60 years[21].

### Sedentary time

There were 7559 participants aged ≥60 in EPIC-Norfolk at the 3^rd^ health check, and a sub-sample of 3784 participants were invited to wear the accelerometer at the time of health check attendance. Participants wore a uniaxial accelerometer (ActiGraph GT1M, ActiGraph, Pensacola, USA) on the right hip for seven days during waking hours except while showering, bathing and swimming. Monitors were initialised to record activity in five-second epochs, which were then integrated into 60-second epochs[22,23]. Non-wear time was defined as continuous zero counts of ≥90 minutes. We only included participants with ≥3 days of valid wear-time (≥600 minutes/day). We excluded participant days with wear-time >18 hours per day (1080 minutes/day) as they were indicative of overnight wear of the accelerometers. Variables derived from accelerometry data included total sedentary time and prolonged sedentary time (sedentary time spent in bouts ≥30 minutes). We defined sedentary behaviour as acceleration <100 counts per minute (cpm)[24–26], an established proxy for measurement of sedentary time[19,20,26–28]. Prolonged sedentary time was defined as consecutive minutes <100 cpm in bouts lasting ≥30 minutes (without interruption). We included descriptive statistics for light physical activity (PA) and MVPA, and defined light PA as acceleration between 100–808 cpm, and MVPA as acceleration ≥809 cpm [29,30].

### Covariates

The sociodemographic factors of interest included age, sex, smoking status (never, former, current), body mass index (BMI; kg/m^2^), social class (Registrar-General's Social Classification; I, II, IIIa, IIIb, IV, V), retirement status (retired, not retired) and education level (further education past 16, no education past 16). The comorbid factors of interest included self-report history of myocardial infarction, stroke, diabetes and cancer. Most of these factors were assessed via a participant completed Health & Lifestyle Questionnaire[31]. BMI was calculated based on weight and height measurements taken by trained research nurses following standard operating procedures.

### Statistical analysis

All analyses were conducted using STATA 15.0 (StataCorp, TX, USA) in June 2019. Although this study has a cross-sectional study design (i.e. the sedentary time, sociodemographic, and comorbid data were collected at a single health check), our analyses examined longitudinal changes in sedentary behaviour over the week that each individual wore the accelerometer. We examined associations between temporally adjacent values of total daily sedentary time (i.e. association between an individual’s sedentary time on an initial day (day d) and their sedentary time on the following day (day d+1)). We also examined associations between temporally adjacent values of time in sedentary bouts ≥30 minutes (i.e. associations between an individual’s prolonged sedentary bouts on an initial day (day d) and their time in these bouts on the following day (day d+1)).

As data were collected for seven consecutive days, each adult provided a maximum of six data points for analysis (e.g. data points for day 1 (d) compared with day 2 (d+1), day 2 compared with day 3, day 3 compared to day 4 etc.). Given that each individual contributed up to seven days of sedentary time, we used mixed multi-level modelling to allow for non-independence of sedentary time between days for each individual. We utilised the xtmixed procedure which is an appropriate technique for multilevel (random coefficients) linear models [32]. Similar methods (generalised linear latent and mixed models, or GLLAMMs) have previously been used to estimate associations between temporally adjacent values, such as pairs of days of sedentary time [12,33]. All models were adjusted for person-level, age, sex and wear-time. We also adjusted models for initial day day-type (weekend vs weekday) and change in day-type (no change, change from weekday to weekend day, change from weekend day to weekday).

The effect estimates (*b*) from the model represented how sedentary time on an initial day was associated with sedentary time on the subsequent day. This meant that every 60 minutes spent sedentary on an initial day (day d) was associated with *b* number of minutes less/more time spent sedentary the following day (day d+1). The *b* output from the original model was for an association per one minute on the initial day. However we reported *b* per 60 minutes on the initial day as the magnitude of values were easier to interpret.

A common misinterpretation of positive correlations between adjacent days (if every additional minute spent in sedentary on day d was associated with *b* minutes more time spent sedentary on day d+1) is that sedentary time would be perpetually higher and higher as time went on. This is not necessarily the case, and depends on the value of the random intercept and whether *b* is greater than one. In order to allow easier interpretation of the models and pre-empt this possibility, we produced line graphs of the model relationships using Microsoft Excel 2010.

We then investigated if these day-to-day associations varied by sociodemographic and comorbid factors specified a priori. These factors included age, sex, smoking status, BMI, social class, retirement status, education level, history of stroke, diabetes, myocardial infarction and cancer. We examined this by adding multiplicative interaction terms into the models one at a time and extracting the *b* coefficients for the interaction, 95% confidence intervals and p values to determine whether any of these variables were significant moderators.

Analyses were done to test whether there were any sociodemographic differences between those that were excluded and those that were included. We also conducted sensitivity analyses to test for differences if we included participants with ≥4 days of valid wear-time versus ≥3 days of valid wear-time, and if we included individual-days with ≥480 minutes/day of valid wear versus ≥600 minutes/day.

## Results

A total of 3459 participants aged ≥60 years were included in our study (Table 1). Of those invited to participate, 18 declined, 16 were excluded due to technical errors with the monitor, 52 were excluded due to insufficient valid monitor wear-time and 194 were excluded due to missing sociodemographic or comorbid data. Of individuals that were included, the mean number of valid accelerometer wear days was 6.71 (SD = 0.68, Range = 3–7). There were no important sociodemographic differences between included and excluded participants.

**Table 1:** Descriptive characteristics of study population stratified by low, medium, and high sedentary time. Sedentary time = ST.

Characteristic		Low ST (≤507 minutes/day) *n = 1152*		Medium ST (508–573 minutes/day) *n = 1148*		High ST (≥574 minutes/day) *n = 1157*	
		Frequency	Percent (%)	Frequency	Percent (%)	Frequency	Percent (%)
**Sex**	**Female**	771	66.9	604	52.6	484	41.8
	**Male**	381	33.1	544	47.4	673	58.2
**Age**	**60–65**	431	37.4	305	26.6	230	19.9
	**65–70**	319	27.7	292	25.4	238	20.6
	**70–75**	247	21.4	269	23.4	246	21.3
	**75–80**	111	9.7	181	15.8	236	20.4
	**80–85**	35	3.0	81	7.1	154	13.3
	**>85**	9	0.8	20	1.7	53	4.5
**Social class**	**I**	88	7.6	99	8.6	124	10.7
	**II**	459	39.9	477	41.6	496	42.9
	**IIIa**	180	15.6	188	16.4	178	15.4
	**IIIb**	244	21.2	247	21.5	218	18.8
	**IV**	157	13.6	117	10.2	118	10.2
	**V**	24	2.1	20	1.7	23	2.0
**Retired**	**Yes**	968	84.0	994	86.6	1021	88.2
	**No**	184	16.0	154	13.4	136	11.8
**Further education after age 16**	**Yes**	513	44.5	490	42.7	530	45.8
	**No**	639	55.5	658	57.3	627	54.2
**Smoking status**	**Current**	40	3.5	36	3.1	34	2.9
	**Former**	489	42.5	526	45.8	600	51.9
	**Never**	623	54.0	586	51.1	523	45.2
**History of stroke**	**No**	1140	99.0	1122	97.7	1120	96.8
	**Yes**	12	1.0	26	2.3	37	3.2
**History of myocardial infarction**	**No**	1124	97.6	1102	96.0	1093	94.5
	**Yes**	28	2.4	46	4.0	64	5.5
**History of diabetes**	**No**	1127	97.9	1108	96.5	1095	94.6
	**Yes**	25	2.1	40	3.5	62	5.4
**History of cancer**	**No**	1025	89.0	1015	88.4	1011	87.4
	**Yes**	127	11.0	133	11.6	146	12.6
**Body Mass Index, kg/m**^**2**^**)**	**<18.5**	13	1.0	6	0.5	5	0.4
	**18.5–25**	495	43.0	368	32.1	304	26.4
	**25–30**	487	42.3	562	49.0	563	48.9
	**30–35**	132	11.5	162	14.1	217	18.8
	**>35**	25	2.2	49	4.3	63	5.5

### Descriptive characteristics

On average, participants were 70.3 years old (SD = 6.8) and were sedentary for 540 mins/day (SD = 80.1, range = 278–846). Mean accelerometer wear time was 834 mins/day (SD = 59.3, range = 611–1014). Participants spent a mean time of 217.1 minutes in light PA (SD = 55.5, range = 16.8–415.1) and a median of 70.5 minutes in MVPA (IQR = 60.4).

Sedentary time was accumulated in bouts of ≥30 minutes for a mean time of 183 mins/day (SD = 87.1, range = 0–641). The total number of “day d to day d+1” comparisons was 19,105. There was a mean difference in total sedentary time between consecutive days of 5.4 minutes (SD = 103). Within-subject standard deviation had a mean of 103 (SD 43.5). There was a mean difference in sedentary time in bouts ≥30 minutes between consecutive days of 1.4 minutes (SD = 114).

### Total sedentary time

Sedentary time on an initial day was associated with sedentary time on the subsequent day (*b* = 9.9 95% CI 9.0, 10.2, p <0.001). On any given day, every 60 minutes spent in sedentary time was associated with 9.9 minutes more time spent sedentary the following day.

**Fig 1:**
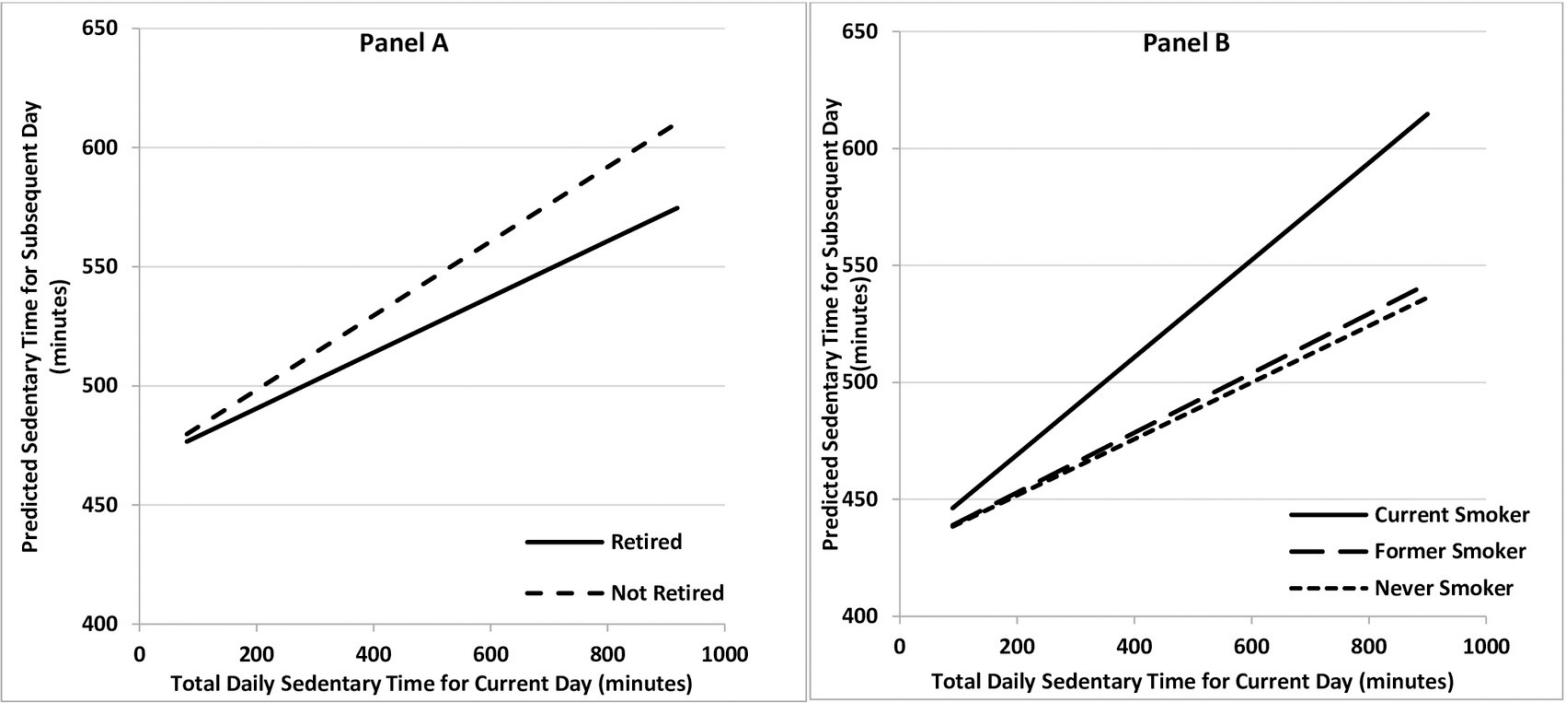
**Association between initial day and subsequent day sedentary time stratified by retirement status (Panel A) and smoking status (Panel B).** Each interaction term was tested in the model separately with all models adjusted for age, sex and wear-time. Values of total daily sedentary time for initial and subsequent day are constrained to the range demonstrated in the dataset (81–919 minutes/day). **Panel A:** Variation by retirement status. Panel A is for 65 year old male. **Panel B:** Variation by smoking status. Panel B is for a 65 year old male.

This association was greater in non-retired compared to retired participants (2.57 extra mins for non-retired versus retired, p = 0.024) and in current smokers compared to former and never-smokers (5.26 extra mins for current versus former, 5.52 extra mins for current versus never, p = 0.023 and 0.017, respectively) (Table 2, Fig 1). It was also greater for those without history of cancer compared with those with a history of cancer (4.57 extra minutes for no history of cancer versus history of cancer, 95% CI 1.94, 7.19). There was no evidence of differential associations for other sociodemographic or comorbid factors.

**Table 2:** Estimated moderation effects of sociodemographic factors on day-to-day associations of sedentary time^a^.

Participant Characteristic		Total Sedentary Time^b^ (minutes/day)			Prolonged Sedentary Time (minutes/day)^c^		
		*b* coefficient	95% CI	p value	*b* coefficient	95% CI	p value
Sex	Female (vs. male)	0.064	-0.66–1.58	1.92	-0.97	-2.61, 0.67	0.25
Age (per year)		0.023	-0.097, 0.14	0.70	**0.18**	**0.061, 0.29**	**0.003**
Retired	No (vs. yes)	**2.57**	**0.34, 4.80**	**0.024**	**-2.74**	**-5.74, -0.21**	**0.034**
Further education after age 16	No (vs. yes)	0.29	-1.01, 1.58	0.98	**1.77**	**0.13, 3.41**	**0.034**
Smoking status	Former (vs. current)	**-5.26**	**-9.78, -0.71**	**0.022**	-0.96	-5.70, 3.77	0.69
	Never (vs. current)	**-5.52**	**-10.1, -0.98**	**0.017**	-2.86	-7.60, 1.88	0.24
Body Mass Index (per kg/m^2^)		0.021	-0.18, 0.22	0.84	-0.033	-0.22, 0.16	0.74
Social Class	II (vs. I)	-1.77	-4.77, 1.22	0.25	**-3.94**	**-6.84, -1.03**	**0.008**
	IIIa (vs. I)	-1.72	-5.12, 1.69	0.34	-2.43	-5.75, 0.89	0.15
	IIIb (vs. I)	-2.72	-5.97, 0.53	0.12	-3.07	-6.26, 0.125	0.060
	IV (vs. I)	0.21	-3.45, 3.87	0.91	-0.081	-4.37, 2.75	0.66
	V (vs. I)	**5.66**	**-0.87, 12.2**	**0.089**	-0.62	-6.86, 5.61	0.85
Stroke	Yes (vs. no)	2.15	-3.65, 9.98	0.47	4.24	-0.72, 9.18	0.094
Myocardial infarction	Yes (vs. no)	2.51	-1.81, 6.82	0.25	-2.83	-6.83, 1.18	0.17
Diabetes	Yes (vs. no)	3.02	-1.40, 7.44	0.18	0.44	-3.64, 4.52	0.83
Cancer	Yes (vs. no)	**-4.57**	**-7.19, -1.94**	**0.0010**	1.41	-4.00, 1.18	0.29

^a^ Estimated moderation effects (*b* coefficients) represent the differences in sedentary time between categories for every additional 60 minutes spent in sedentary on an initial day compared to the subsequent day.

All models adjusted for age, sex and wear-time. Boldface is used for significant interactions.

^b^ Every additional 60 minutes spent in sedentary on an initial day was associated with *b* minutes less/more time spent sedentary on the subsequent day for the category compared to the reference (in brackets).

^c^ Every additional 60 minutes spent in prolonged sedentary bouts on an initial day was associated with *b* minutes less/more time spent in these bouts on the subsequent day for the categories compared to the reference.

### Prolonged sedentary bouts

**Fig 2:**
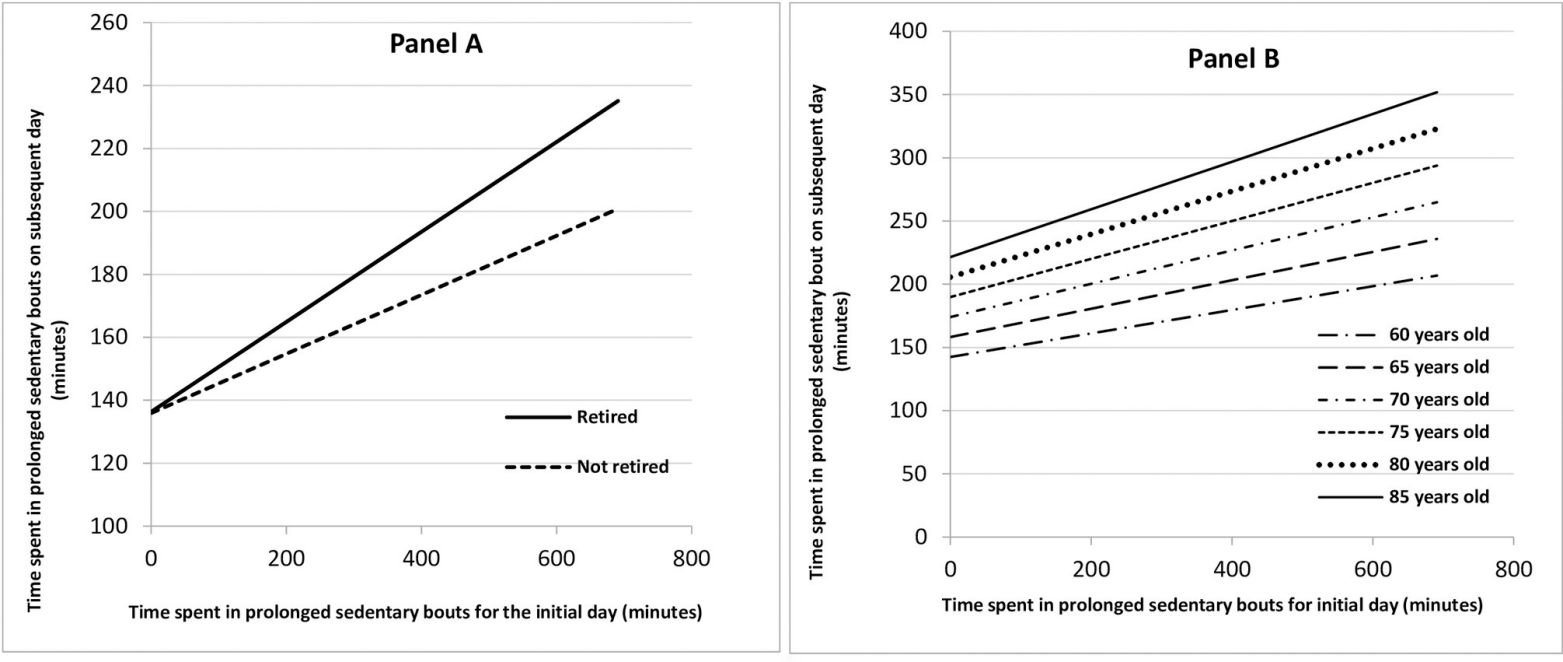
**Association between initial day and subsequent day time in prolonged sedentary bouts by retirement status (Panel A) and by age (Panel B).** Each interaction term was tested in the model separately with all models adjusted for age, sex and wear-time. Values of time spent in sedentary bouts ≥30 minutes for the initial day are constrained to the range within the dataset (0–691 minutes/day). **Panel A**: Variation by retirement status. Panel A is for a 65 year old male. **Panel B:** Variation by age. Panel B is for a male.

Time spent in prolonged sedentary bouts (≥30 minutes) on an initial day was positively associated with time spent in these bouts on the subsequent day (*b* = 7.8, 95% CI 7.2, 8.4, p<0.001). On any given day, every 60 minutes spent in prolonged sedentary bouts was associated with 7.8 minutes more time spent in these bouts the following day. This association was greater in older individuals (0.18 extra mins per year of age, 95% CI 0.061, 0.29), and in retired compared to non-retired participants (2.74 extra mins for retired compared to non-retired, 95% CI 0.21, 5.74) (Fig 2). There was no evidence of differential associations for other sociodemographic or comorbid factors.

### Sensitivity analyses

There were no significant differences if we included participants with ≥4 days of valid wear-time versus ≥3 days of valid wear-time, and no differences if we included individual-days with ≥480 minutes/day of valid wear versus ≥600 minutes/day. There was no material difference in terms of direction, size and statistical significance to our findings after adjustment for initial day day-type and change in day-type.

## Discussion

Here we report that the amount of time spent sedentary on an initial day is related to the time spent sedentary on a subsequent day among a cohort of older UK adults. This means that the more time an individual spends being sedentary on an initial day, the greater the subsequent day’s sedentary time is. Importantly, this positive effect is small. An individual’s daily sedentary time therefore appears to be made up of a large, reasonably stable component (demonstrated by the constant in the model, which is the intersept of the Y-axis in Figs 1 and 2) and a small component positively dependent on the previous day’s behaviour. We found a similar result for prolonged sedentary bouts.

These findings might be explained in the context of the social-psychological model of dual processing theory[34]. Spending time being sedentary is preceded by a decision to be sedentary. In the model of dual processing, the decision to be sedentary may be reached through a habitual, ‘automatic response’ (fast decision with minimal thought required), or an ‘intentional response’ (a rational and slower decision). Habitual responses are defined as those generated “though automatic responses to contextual cues acquired through repetition of behaviour in the presence of these cues”[35,36]. Given that individuals demonstrate a large stable component, one explanation is that older adults may be demonstrating that they are largely habitual in their sedentary behaviour with a small capacity for variation though intentional responses [37].

Thus, it may be that most decisions to sit during the day are habitually cued by the environment (e.g. sitting in front of the television at home, entering the office and sitting at the desk), and are favoured due to the ease and speed with which they can be performed compared to alternatives. ‘Habitual’ sedentary time plausibly would take up a large, stable proportion of total daily sedentary time given that especially as we age contextual cues tend to be less varying (e.g. same house, same shops).

Concurrently, it is intuitive that a much smaller proportion of decisions to sit might be conscious e.g sitting or standing on the bus when there are only a few seats available. These thoughtful decisions may be a much smaller proportion of total sedentary time, may lead to variation from day-to-day and may positively impact on the next day’s sedentary behaviour as part of future habit formation. The dual process model suggests that there is a habit formation period as behaviour is repeated in the same context and behaviour control gradually moves from being directed by beliefs, attitudes, and intention to being triggered by contextual cues [38,39].

Thus, the small positive associations we observed occurring day-to-day may be a demonstration of the process of habit formation. Alternatively, it might be a consequence of the nature of the micro-environment of consecutive days e.g. I didn’t go out the last two days because it was colder so sat and watched TV more.

Our findings are important as habitual behaviours are settled or regular tendencies in associated contexts, which often makes them hard to change and overrule counter-habitual intentions [36,39,40].

Future interventions ought to consider how we might target changing habitual sedentariness (automatic responses), and potentially replace it with another habit[39,41]. If we can target these automatic responses by, for example, a monitoring device alerting us to a prolonged bout of sitting, we may be able to trigger an intentional response. As context is important in driving habits, changing the context could be a target of future interventions by for example either removing the person from the environment that cues unwanted habitual responses or by modifying the context, e.g., placing reminders in the environment[35,42–44].

Our findings do not support the theory of day-to-day sedentary time compensation in older adults. We cannot, however, rule out within-day compensation or compensation over a longer period. Our findings means that individuals are capable of varying their time being sedentary by small amounts, and future interventions may be able to capitalise on this by encouraging initial change. Because a large proportion of total sedentary time is stable, this may suggest that if we can create a shift in time spent sedentary with an intervention the habit may help it perpetuate.

Day-to-day associations in total sedentary time were greatest in non-retired adults, in current smokers, and in those without a history of cancer This suggests that these sub-groups display greater day-to-day variability in sedentary time and may therefore have less consolidated habits and might be more amenable to behaviour change. If this is the case, they may represent important targets for intervention. Conversely, these same groups may present the opposite challenge for maintenance of new habits. Day-to-day variability seemed largely unaffected by comorbid status.

It is intuitive that smokers demonstrate more variability in total sedentary time than their non-smoking counterparts. Smoking may represent a non-sedentary activity and smokers may vary in the number of cigarettes and timing of smoking from day-to-day due to changes in location (e.g. moving from non-smoking public places to standing outside). Given that smokers are likely to be having regular smoking breaks, replacing this behaviour with other another type of break might be a good basis for a joint smoking/sedentary time intervention. It is also intuitive that non-retired adults may have more variability, given that their routines may look different from day-to-day depending on their job and whether it is a working or non-working day. This bolsters the case for targeting the transition to retirement as a time to modify sedentary behaviour[45].

We also found that the amount of time spent in prolonged sedentary bouts on an initial day was related to the time spent in these bouts on a subsequent day and that day-to-day associations of time in prolonged sedentary bouts was greatest in older individuals and the retired. Why this is the case is unclear. This finding suggests that the retired and older adults might be less resistant to initial behaviour change in prolonged sedentary bouts.

A systematic review [11] in adults reported that in 11 out of 28 studies, including two studies in older adults, physical activity or energy expenditure compensation occurred after an imposed exercise stimulus. In older adults, early evidence supports compensation for increased physical activity after exercise intervention with reduced spontaneous physical activity[15,46,47] and reduced or no change in energy expenditure[48–51] at a time weeks to months later. In spite of this, sedentary time compensation has largely been unscrutinised even in the general adult population. Donaldson et al. [52] (n = 293) reported that sedentary time appears to be stable from day-to-day in a USA sample of adults (mean age = 55±14 years) although this comparison was between days of the week rather than between consecutive days.

Our study is the first to examine whether day-to-day sedentary time compensation occurs in older adults. Similar analyses examining day-to-day sedentary time compensation have so far only been reported in children, showing support for within-day but not between-day compensation [12,33]. Additionally, this is the first analysis to our knowledge, to report on day-to-day time in prolonged sedentary bouts. Given that prolonged sedentary bouts are thought to be particularly deleterious [6–9] and older adults accumulate significant proportions of sedentary time in prolonged bouts[53,54], it is important to understand day-to-day variability of prolonged bouts so we can more successfully target it.

### Strengths & limitations

Our study has several key strengths. EPIC-Norfolk is a well-characterised, large population-based cohort with well-documented sampling methods and use of objective measures of sedentary time. However, EPIC-Norfolk participants represent a slightly healthier sample than the general UK population with limited ethnic diversity [55] leading to the potential of healthy volunteer bias. We had high levels of participation among those who were invited to wear accelerometers and a high level of complete data.

Our accelerometry data processing decisions (<100cpm threshold with the use of 60-second epochs) allow easy comparison with the existing literature. However, accelerometers have several well documented limitations. They do not provide information on body posture and so may misclassify standing still as being sedentary[56] and they primarily measure movement when worn over the hip, and so miss upper body movement. The ActiGraph GT1M accelerometer may overestimate sedentary breaks, and therefore underestimate sedentary bouts, in comparison to the other accelerometers such as ActivPal (device worn on the thigh that uses information about static and dynamic acceleration to distinguish body posture as sitting/lying and standing and stepping)[28,57,58].

The wear protocol included instructions for participants to take off monitors for sleep and water-based activities. Any such non-wear activities may be misclassified as sedentary time. However, the non-wear algorithm defined a non-wear time threshold of ≥90 minutes. Evidence supports the 90-minute threshold as a good estimate of non-wear bouts per day [24,59,60]. Further, it has been suggested that people wearing accelerometers may demonstrate Hawthorne-like effects (i.e. their behaviours may change as a result of being measured) [61,62].

It is possible that compensation may be occurring such that a decrease in sedentary time on an initial day may lead to compensatory effects on light physical activity or MVPA. Future analyses examining cross-compensation with other behaviours may be warranted. Further, it is possible that sedentary time compensation exists above a threshold, and therefore we did not observe such compensation. It will be important for future randomised control trials undertaking sedentary behaviour interventions to examine whether they cross any such threshold and illicit compensatory change.

We used an observational design to examine whether sedentary compensation could be triggered by naturally occurring day-to-day fluctuations, but it will be important for future studies to examine whether compensation occurs following planned experimental intervention.

## Conclusions

In this study on sedentary patterns, we found that all day-to-day associations were positive, which is indicative of a habitual rather than a random pattern of behaviour. Day-to-day sedentary time variability may reflect individuals’ capability to change behaviour. There are notable differences for total and prolonged sedentary behaviour patterns. Though non-retired individuals display larger positive day-to-day associations for total sedentary time compared to retired individuals, they display smaller day-to-day positive associations for prolonged bouts. If subgroups with greater day-to-day variability are more amenable to behaviour change, pre-retirement interventions might be more effective if they target total sedentary time and post-retirement interventions might be more effective if they target prolonged sedentary bouts. As some sub-groups are more variable in their behaviours (i.e. their habits appear less consolidated), we may be able to utilise this knowledge to tailor interventions and to target changes in sedentary behaviours. We have added to this under-researched area by demonstrating that older adults demonstrate positive habitual patterning in their sedentary behaviours but do not exhibit day-to-day sedentary time compensation.

## References

[1] Schmid D, Ricci C, Leitzmann MF. Associations of objectively assessed physical activity and sedentary time with all-cause mortality in US adults: the NHANES study. PLoS One. 2015;10: e0119591 10.1371/journal.pone.0119591 25768112 PMC4358950

[2] Koster A, Caserotti P, Patel K V., Matthews CE, Berrigan D, Van Domelen DR, et al Association of Sedentary Time with Mortality Independent of Moderate to Vigorous Physical Activity. Ruiz JR, editor. PLoS One. 2012;7: e37696 10.1371/journal.pone.0037696 22719846 PMC3374810

[3] Brocklebank LA, Falconer CL, Page AS, Perry R, Cooper AR. Accelerometer-measured sedentary time and cardiometabolic biomarkers: A systematic review. Prev Med (Baltim). 2015;76: 92–102. 10.1016/j.ypmed.2015.04.013 25913420

[4] Patterson R, McNamara E, Tainio M, de Sá TH, Smith AD, Sharp SJ, et al Sedentary behaviour and risk of all-cause, cardiovascular and cancer mortality, and incident type 2 diabetes: a systematic review and dose response meta-analysis. Eur J Epidemiol. 2018 [cited 22 Jul 2018]. 10.1007/s10654-018-0380-1 29589226 PMC6133005

[5] Harvey JA, Chastin SFM, Skelton DA. How Sedentary Are Older People? A Systematic Review of the Amount of Sedentary Behavior. J Aging Phys Act. 2015;23: 471–487. 10.1123/japa.2014-0164 25387160

[6] Dunstan DW, Kingwell BA, Larsen R, Healy GN, Cerin E, Hamilton MT, et al Breaking up prolonged sitting reduces postprandial glucose and insulin responses. Diabetes Care. 2012;35: 976–83. 10.2337/dc11-1931 22374636 PMC3329818

[7] Saunders TJ, Larouche R, Colley RC, Tremblay MS. Acute sedentary behaviour and markers of cardiometabolic risk: a systematic review of intervention studies. J Nutr Metab. 2012;2012: 712435 10.1155/2012/712435 22754695 PMC3382951

[8] Diaz KM, Howard VJ, Hutto B, Colabianchi N, Vena JE, Safford MM, et al Patterns of Sedentary Behavior and Mortality in U.S. Middle-Aged and Older Adults. Ann Intern Med. 2017;167: 465 10.7326/M17-0212 28892811 PMC5961729

[9] Henson J, Yates T, Biddle SJH, Edwardson CL, Repository I, Henson J, et al Associations of objectively measured sedentary behaviour and physical activity with markers of cardiometabolic health. Diabetologia. 2013;56: 1012–20. 10.1007/s00125-013-2845-9 23456209

[10] Shrestha N, Grgic J, Wiesner G, Parker A, Podnar H, Bennie JA, et al Effectiveness of interventions for reducing non-occupational sedentary behaviour in adults and older adults: a systematic review and meta-analysis. Br J Sports Med. 2018; bjsports-2017-098270. 10.1136/bjsports-2017-098270 29331992

[11] Gomersall SR, Rowlands A V, English C, Maher C, Olds TS. The ActivityStat Hypothesis The Concept, the Evidence and the Methodologies. 2012 [cited 1 Feb 2019]. 10.1007/s40279-012-0008-7 23329607

[12] Ridgers ND, Barnett LM, Lubans DR, Timperio A, Cerin E, Salmon J. Potential moderators of day-to-day variability in children’s physical activity patterns. J Sports Sci. 2018;36: 637–644. 10.1080/02640414.2017.1328126 28532318

[13] Garland T, Schutz H, Chappell MA, Keeney BK, Meek TH, Copes LE, et al The biological control of voluntary exercise, spontaneous physical activity and daily energy expenditure in relation to obesity: human and rodent perspectives. J Exp Biol. 2011;214: 206–229. 10.1242/jeb.048397 21177942 PMC3008631

[14] Thorburn AW, Proietto J. Biological determinants of spontaneous physical activity. Obes Rev. 2000;1: 87–94. Available: http://www.ncbi.nlm.nih.gov/pubmed/12119990 12119990 10.1046/j.1467-789x.2000.00018.x

[15] Gray P, Murphy M, Gallagher A, Simpson EEA. A qualitative investigation of physical activity compensation among older adults. Br J Health Psychol. 2018;23: 208–224. 10.1111/bjhp.12282 29171704

[16] Harvey JA, Chastin SFM, Skelton DA. Prevalence of sedentary behavior in older adults: a systematic review. Int J Environ Res Public Health. 2013;10: 6645–61. 10.3390/ijerph10126645 24317382 PMC3881132

[17] Hayat SA, Luben R, Keevil VL, Moore S, Dalzell N, Bhaniani A, et al Cohort profile: A prospective cohort study of objective physical and cognitive capability and visual health in an ageing population of men and women in Norfolk (EPIC-Norfolk 3). Int J Epidemiol. 2014;43: 1063–1072. 10.1093/ije/dyt086 23771720 PMC4121549

[18] Day N, Oakes S, Luben R, Khaw KT, Bingham S, Welch A, et al EPIC-Norfolk: study design and characteristics of the cohort. European Prospective Investigation of Cancer. Br J Cancer. 1999;80 Suppl 1: 95–103. Available: http://www.ncbi.nlm.nih.gov/pubmed/10466767 10466767

[19] Copeland JL, Esliger DW. Accelerometer assessment of physical activity in active, healthy older adults. J Aging Phys Act. 2009;17: 17–30. 19299836 10.1123/japa.17.1.17

[20] Kozey-Keadle S, Libertine A, Lyden K, STAUDENMAYER J, Freedson PS. Validation of wearable monitors for assessing sedentary behavior. Med Sci Sports Exerc. 2011;43: 1561–7. 10.1249/MSS.0b013e31820ce174 21233777

[21] World Health Organisation. WHO | Ageing and life-course In: Ageing and lifecourse [Internet]. World Health Organization; 2013 [cited 12 Sep 2018]. Available: http://www.who.int/ageing/en/

[22] Ojiambo R, Cuthill R, Budd H, Konstabel K, Casajús JA, González-Agüero A, et al Impact of methodological decisions on accelerometer outcome variables in young children. Int J Obes (Lond). 2011;35 Suppl 1: S98–S103. 10.1038/ijo.2011.40 21483428

[23] Edwardson CL, GORELY T. Epoch length and its effect on physical activity intensity. Med Sci Sports Exerc. 2010;42: 928–934. 10.1249/MSS.0b013e3181c301f5 19996997

[24] Matthews CE, George SM, Moore SC, Bowles HR, Blair A, Park Y, et al Amount of time spent in sedentary behaviors and cause-specific mortality in US adults. Am J Clin Nutr. 2012;95: 437–45. 10.3945/ajcn.111.019620 22218159 PMC3260070

[25] Ridgers ND, Salmon J, Ridley K, O’Connell E, Arundell L, Timperio A. Agreement between activPAL and ActiGraph for assessing children’s sedentary time. Int J Behav Nutr Phys Act. 2012;9: 15 10.1186/1479-5868-9-15 22340137 PMC3311087

[26] Aguilar-Farías N, Brown WJ, Peeters GMEE Geeske MEEG. ActiGraph GT3X+ cut-points for identifying sedentary behaviour in older adults in free-living environments. J Sci Med Sport. 2014;17: 293–9. 10.1016/j.jsams.2013.07.002 23932934

[27] Lee I, Shiroma EJ. Using accelerometers to measure physical activity in large-scale epidemiological studies: issues and challenges. Br J Sports Med. 2014;48: 197–201. 10.1136/bjsports-2013-093154 24297837 PMC3947179

[28] Lyden K, Kozey Keadle SL, Staudenmayer JW, Freedson PS. Validity of Two Wearable Monitors to Estimate Breaks from Sedentary Time. Med Sci Sport Exerc. 2012;44: 2243–2252. 10.1249/MSS.0b013e318260c477 22648343 PMC3475768

[29] Hall KS, Howe CA, Rana SR, Martin CL, Morey MC. METs and accelerometry of walking in older adults: Standard versus measured energy cost. Med Sci Sports Exerc. 2013;45: 574–582. 10.1249/MSS.0b013e318276c73c 23059862 PMC5822436

[30] Ried-Larsen M, Brønd JC, Brage S, Hansen BH, Grydeland M, Andersen LB, et al Mechanical and free living comparisons of four generations of the Actigraph activity monitor. Int J Behav Nutr Phys Act. 2012;9: 113 10.1186/1479-5868-9-113 22971175 PMC3463450

[31] MRC Epidemiology. EPIC-Norfolk: health check and laboratory protocol. 2015 [cited 12 Sep 2018]. Available: http://www.srl.cam.ac.uk/epic/about/protocols.shtml

[32] Stata. xtmixed: Multilevel mixed-effects linear regression. Stata 12 User’s guide 2012 pp. 302–354. doi:S096098220300575X [pii]

[33] Ridgers ND, Timperio A, Cerin E, Salmon J. Within- and between-day associations between children’s sitting and physical activity time. BMC Public Health. 2015;15 10.1186/s12889-015-2291-3 26400793 PMC4581512

[34] Houlihan S. Dual-process models of health-related behaviour and cognition: a review of theory. Public Health. 2018;156: 52–59. 10.1016/j.puhe.2017.11.002 29408189

[35] Nilsen P, Roback K, Broström A, Ellström E. Creatures of habit: accounting for the role of habit in implementation research on clinical behaviour change. 2012 Available: http://www.implementationscience.com/content/7/1/53 10.1186/1748-5908-7-53 PMC3464791 22682656

[36] Wood W, Unger DR¨. Psychology of Habit. 2015 [cited 4 Sep 2019]. 10.1146/annurev-psych-122414-033417 26361052

[37] Maher JP, Conroy DE. A dual-process model of older adults’ sedentary behavior. Heal Psychol. 2016;35: 262–272. 10.1037/hea0000300 26690644

[38] Lally P, Wardle J, Gardner B. Experiences of habit formation: A qualitative study. Psychol Health Med. 2011;16: 484–489. 10.1080/13548506.2011.555774 21749245

[39] Gardner B. A review and analysis of the use of “habit” in understanding, predicting and influencing health-related behaviour. Health Psychol Rev. 2014;0. 10.1080/17437199.2013.876238 25207647 PMC4566897

[40] Hall PA, Fong GT. Temporal self-regulation theory: A model for individual health behavior. Health Psychol Rev. 2007;1: 6–52. 10.1080/17437190701492437

[41] Rothman AJ, Sheeran P, Wood W. Reflective and Automatic Processes in the Initiation and Maintenance of Dietary Change. Ann Behav Med. 2009;38: 4–17. 10.1007/s12160-009-9130-7 19787308

[42] Bamberg S. Is a Residential Relocation a Good Opportunity to Change People’s Travel Behavior? Results From a Theory-Driven Intervention Study. Environ Behav. 2006;38: 820–840. 10.1177/0013916505285091

[43] Heatherton TF, Nichols PA. Personal Accounts of Successful Versus Failed Attempts at Life Change. Personal Soc Psychol Bull. 1994;20: 664–675. 10.1177/0146167294206005

[44] Wood W, Tam L, Witt MG. Changing circumstances, disrupting habits. J Pers Soc Psychol. 2005;88: 918–933. 10.1037/0022-3514.88.6.918 15982113

[45] Sprod J, Ferrar K, Olds T, Maher C. Changes in sedentary behaviours across the retirement transition: A systematic review. Age and Ageing. 2015 pp. 918–925. 10.1093/ageing/afv140 26504115

[46] Di Blasio A, Ripari P, Bucci I, Di Donato F, Izzicupo P, D’Angelo E, et al Walking training in postmenopause. Menopause J North Am Menopause Soc. 2012;19: 23–32. 10.1097/gme.0b013e318223e6b3 21993080

[47] Di Blasio A, Di Donato F, Di Santo S, Bucci I, Izzicupo P, Di Baldassarre A, et al Aerobic physical exercise and negative compensation of non-exercise physical activity in post-menopause: a pilot study. J Sports Med Phys Fitness. 2018;58: 1497–1508. 10.23736/S0022-4707.17.07320-0 28597615

[48] Hunter GR, Bickel CS, Fisher G, Neumeier WH, Mccarthy JP. Combined Aerobic and Strength Training and Energy Expenditure in Older Women. Med Sci Sport Exerc. 2013;45: 1386–1393. 10.1249/MSS.0b013e3182860099 23774582 PMC3713080

[49] Goran MI, Poehlman ET. Endurance training does not enhance total energy expenditure in healthy elderly persons. Am J Physiol Metab. 1992;263: E950–E957. 10.1152/ajpendo.1992.263.5.E950 1443128

[50] Mcneil J, Brenner DR, Courneya KS, Friedenreich CM. Dose–response effects of aerobic exercise on energy compensation in postmenopausal women: combined results from two randomized controlled trials. Int J Obes. 2017;41: 1196–1202. 10.1038/ijo.2017.87 28360432 PMC5550560

[51] Wang X, Bowyer KP, Porter RR, Breneman CB, Custer SS. Energy expenditure responses to exercise training in older women. Physiol Rep. 2017;5 10.14814/phy2.13360 28774950 PMC5555889

[52] Donaldson SC, Montoye AHK, Tuttle MS, Kaminsky LA. Variability of Objectively Measured Sedentary Behavior. Med Sci Sports Exerc. 2016;48: 755–61. 10.1249/MSS.0000000000000828 26606270

[53] Jefferis BJ, Sartini C, Shiroma E, Whincup PH, Wannamethee SG, Lee I-M. Duration and breaks in sedentary behaviour: accelerometer data from 1566 community-dwelling older men (British Regional Heart Study). Br J Sports Med. 2014;49: 1591–1594. 10.1136/bjsports-2014-093514 25232029 PMC4363289

[54] Shiroma EJ, Freedson PS, Trost SG, Lee I. Patterns of accelerometer-assessed sedentary behavior in older women. Jama. 2013;310: 2562–2563. 10.1001/jama.2013.278896 24346993 PMC3869030

[55] Fry A, Littlejohns TJ, Sudlow C, Doherty N, Adamska L, Sprosen T, et al Comparison of Sociodemographic and Health-Related Characteristics of UK Biobank Participants With Those of the General Population. Am J Epidemiol. 2017;186: 1026–1034. 10.1093/aje/kwx246 28641372 PMC5860371

[56] Bassett DR, John D, Conger SA, Rider BC, Passmore RM, Clark JM. Detection of lying down, sitting, standing, and stepping using two activPAL monitors. Med Sci Sports Exerc. 2014;46: 2025–2029. 10.1249/MSS.0000000000000326 24598698

[57] Barreira T V., Zderic TW, Schuna JM, Hamilton MT, Tudor-Locke C. Free-living activity counts-derived breaks in sedentary time: Are they real transitions from sitting to standing? Gait Posture. 2015;42: 70–72. 10.1016/j.gaitpost.2015.04.008 25953504

[58] Júdice PB, Santos DA, Hamilton MT, Sardinha LB, Silva AM. Validity of GT3X and Actiheart to estimate sedentary time and breaks using ActivPAL as the reference in free-living conditions. Gait Posture. 2015;41: 917–922. 10.1016/j.gaitpost.2015.03.326 25852024

[59] Choi L, Liu Z, Matthews CE, Buchowski MS. Validation of accelerometer wear and nonwear time classification algorithm. Med Sci Sports Exerc. 2011;43: 357–364. 10.1249/MSS.0b013e3181ed61a3 20581716 PMC3184184

[60] Mailey EL, Gothe NP, Wójcicki TR, Szabo AN, Olson EA, Mullen SP, et al Influence of allowable interruption period on estimates of accelerometer wear time and sedentary time in older adults. J Aging Phys Act. 2014;22: 255–260. 10.1123/japa.2013-0021 23752299 PMC3875619

[61] Clemes SA, Deans NK. Presence and duration of reactivity to pedometers in adults. Med Sci Sports Exerc. 2012;44: 1097–1101. 10.1249/MSS.0b013e318242a377 22595985

[62] Clemes SA, Parker RAA. Increasing our understanding of reactivity to pedometers in adults. Med Sci Sports Exerc. 2009;41: 674–680. 10.1249/MSS.0b013e31818cae32 19204581

